# Supplementary material for: Effects of Long-Term Nitrogen Fertilization on the Formation of Metabolites Related to Tea Quality in Subtropical China
Source: Metabolites. 2021 Mar 2;11(3):146. doi: 10.3390/metabo11030146 (PMC8000315; doi:10.3390/metabo11030146)
Supplement: Supplementary file 1 [file metabolites-11-00146-s001.pdf]

# SUPPLEMENTARY INFORMATION

# Effects of Long-Term Nitrogen Fertilization on the Formation of Metabolites Related to Tea Quality in Subtropical China

**Yuzhen Chen [1,2,†], Feng Wang [1,2,†], Zhidan Wu [1,2], Fuying Jiang [1,2], Wenquan Yu [3], Jie Yang [4], Jiaming Chen [4], Guotai Jian [4], Zhiming You [1,2,*] and Lanting Zeng [4,*]**

[1] Tea Research Institute, Fujian Academy of Agricultural Sciences, No. 104 Pudang Road, Xindian Town, Jin'an District, Fuzhou 350012, China; taotaoyuzhen@163.com (Y.C.); 82458lin@163.com (F.W.); wzd@163.com (Z.W.); fuyingjiang93@163.com (F.J.)

[2] National Agricultural Experimental Station for Soil Quality, No. 1 Hutouyang Road, Shekou Town, Fu'an 355015, Fujian, China

[3] Fujian Academy of Agricultural Sciences, No. 247 Wusi Road, Gulou District, Fuzhou 350013, China; ywq1972@163.com (W.Y.)

[4] Key Laboratory of South China Agricultural Plant Molecular Analysis and Genetic Improvement & Guangdong Provincial Key Laboratory of Applied Botany, South China Botanical Garden, Chinese Academy of Sciences, No. 723 Xingke Road, Tianhe District, Guangzhou 510650, China; yangjie0727@163.com (J.Y.); chenjiaming@scbg.ac.cn (J.C.); jiangt@scbg.ac.cn (G.J.)

* Correspondence: youzm1964@163.com (Z.Y.); zenglanting@scbg.ac.cn (L.Z.), Tel.: +86-20-3702-1938

† These authors contributed equally to this work.

**Table S1.** The primers used for quantitative real time PCR (qRT-PCR) in the study.

| Gene | Accession number | Forward primer 5'-3' | Reverse primer 5'-3' |
|---|---|---|---|
| *CsEF1-α* | KA280301.1 | TTGGACAAGCTCAAGGCTGAACG | ATGGCCAGGAGCATCAATGACAGT |
| *CsHEMA1* | XM_028228202.1 | TGCAGCTGACAGGTATACAAA | ATGGCAAGCTTTTCACGCATT |
| *CsHEMA2* | - | ATGGCAAGCTTTTCACGCATT | ATGGCAAGCTTTTCACGCATT |
| *CsPOR1* | XM_028215906.1 | GGCTCCATAACAGGAAACACA | TTCAAGCCTCCTGCAAGTCC |
| *CsPOR2* | XM_028228181.1 | AGGGAAAGCCTAGTGTTTCTTTGA | GCCATTGGTTTGATTCCTTCTTGA |
| *CsPAO* | XM_028208343.1 | TCGCTCCTCTCTCTGAAGGT | AGGCCTGAGGGATTCGAGTA |
| *CsTS1* | TEA015198.1 | GTTGATGTTTCTGGGCAGCA | CTCACCCACACCAGTCAGAT |
| *CsGS1.1* | MG778703 | CAGCACCAAGTCTACGAGGA | AATCATGGAAGTAACCACA |
| *CsGS1.2* | MG778705 | TGCAAACCGTGGTGCATCTG | GTTTCCACAGGATGGTGGTAG |
| *CsGS1.3* | MG778704 | TGGCCGATTGGTTGGCCTG | ACAACTGATCTCCAGAACT |
| *CsGS2* | MG778706 | CTGGAACGGTGCAGGATGC | GCCCCACGCGGATTGAACA |
| *CsPDX2.1* | MT726050 | AAGGAAACTTGCTAGGGACTGC | CAACAATGCTGCTACTTGAGGC |
| *CsPAL* | D26596 | ATTCCTTGCCAATCCTGTAA | ACTGCCTCGGCTGTCTTTCT |
| *CsAAAT1* | MH544095 | CGCCGACGAACATCACAATC | TGGGTCTCCCATACCCAGAG |
| *CsAAAT2* | MH544096 | CCAAGGGAACAAAGGGCTGA | CCGTTGAATTTGGCGGATCG |
| *CsNES* | KY033151 | CAGCACAAACGAAATTTCCT | CATTCCATGACCCAAGAGAA |
| *CsTSA* | KX022968 | ACCACACCTACTACTCCAACA | CTTACAGATACACGAGCACCAG |
| *CsTSB2* | KX022970 | CCTTATCTCCACGCCCACTA | ACGACTATGCCGACTTGAAG |
| *CsLOX1* | EU195885 | GCTGACTGGACAACCGATGA | CAACATATGCTTCTATGAAAATGC |
| *CsMYC2a* | KU892079 | ATCCCGGTTTTCAGGTCCAC | ATTCGAATCATCGCGTCCCA |
| *CsMYC2b* | KU892080 | TTGCCCTTTGGATACCCACC | TTCGCGTGAAAATGCTGCAA |
| *CsMYC2c* | KU892081 | TGCAACAAGCCAAGTCACTG | AGCTCAGATTCGGCATTGGT |

*EF1-α, encoding elongation factor 1-α; HEMA, glutamyl-tRNA reductase; POR, protochlorophyllide oxidoreductase; PAO, pheophorbide a oxygenase; TS, L-theanine synthase; GS, L-glutamine synthase; PDX, pyridoxal 5′-phosphate synthase subunit; PAL, phenylalanine lyase; AAAT, aromatic amino acid aminotransferase; NES, (E)-nerolidol synthase; TSA, tryptophan synthase α-subunit; TSB, tryptophan synthase β-subunit; LOX, lipoxygenase; PPDC, phenylpyruvate decarboxylase; MYC, myelocytomatosis protein.*

**Table S2.** Effects of nitrogen application on the contents of aroma compounds in fresh tea leaves collected in spring.

| Aroma compound (nmol/g FW) | N0 | N1 | N2 | N3 |
|---|---|---|---|---|
| Benzyl alcohol | 25.85±3.50a | 22.71±4.27ab | 18.24±3.23bc | 16.11±2.99c |
| Phenylethanol | 88.77±8.91a | 80.60±19.99a | 57.53±11.12b | 55.15±5.97c |
| Methyl salicylate | 25.16±4.53a | 25.49±3.06a | 20.13±2.52b | 18.89±0.86b |
| Geraniol | 502.25±61.82a | 482.68±99.80a | 347.12±59.92b | 313.18±29.59b |
| 1-Hexanol | 1.23±0.17a | 1.03±0.47a | 0.91±0.15a | 0.90±0.29a |
| Benzaldehyde | 0.36±0.08a | 0.35±0.047a | 0.34±0.09a | 0.37±0.11a |
| Phenylacetaldehyde | 1.24±0.46a | 1.13±0.27a | 1.12±0.18a | 1.70±0.50a |
| (*E*)-Nerolidol | 0.90±0.17a | 0.86±0.16a | 1.12±0.30a | 0.78±0.11a |

| | | | | |
|---|---|---|---|---|
| Linalool | 147.68±22.31a | 140.97±34.72a | 117.56±15.22a | 110.89±16.45a |
| Linalool oxide I | 24.33±4.95a | 28.27±3.48a | 23.67±1.02a | 22.65±4.63a |
| Linalool oxide II | 71.02±13.17a | 84.86±9.41a | 72.23±2.64a | 70.75±12.71a |
| Hexanal | 5.44±0.63a | 5.78±1.35a | 5.40±1.73a | 5.26±0.96a |
| *cis*-3-Hexenyl acetate | 3.18±0.50a | 2.86±0.92a | 2.83±1.22a | 2.24±0.17a |
| Indole | 0.22±0.12a | 0.12±0.04a | 0.11±0.08a | 0.09±0.01a |

Values followed by different letters (a–c) mean significant at the 5% level among different treatments.
